# Platelet-Rich Plasma (PRP): Molecular Mechanisms, Actions and Clinical Applications in Human Body

**DOI:** 10.3390/ijms262110804

**Published:** 2025-11-06

**Authors:** Wen-Shan Wu, Li-Ru Chen, Kuo-Hu Chen

**Affiliations:** 1 Department of Medical Education, Taipei Tzu-Chi Hospital, The Buddhist Tzu-Chi Medical Foundation, New Taipei City 231, Taiwan; amy142417aa@gmail.com; 2 Department of Physical Medicine and Rehabilitation, Mackay Memorial Hospital, Taipei 104, Taiwan; gracealex168@gmail.com; 3 Department of Mechanical Engineering, National Yang Ming Chiao Tung University, Hsinchu 300, Taiwan; 4 Department of Obstetrics and Gynecology, Taipei Tzu-Chi Hospital, The Buddhist Tzu-Chi Medical Foundation, New Taipei City 231, Taiwan; 5 School of Medicine, Tzu-Chi University, Hualien 970, Taiwan

**Keywords:** platelet-rich plasma, platelets releasing growth factors, PRP, VEGF, PDGF

## Abstract

Platelet-rich plasma (PRP) is an autologous blood-derived concentrate increasingly utilized in regenerative medicine for its ability to accelerate healing and tissue repair. PRP is broadly classified by leukocyte content, fibrin architecture, and platelet concentration, with classification systems developed to standardize characterization. Preparation methods, including single- or double-spin centrifugation and buffy coat techniques, influence the final composition of PRP, determining the relative proportions of platelets, leukocytes, plasma proteins, and extracellular vesicles. These components act synergistically, with platelets releasing growth factors (e.g., VEGF, PDGF, TGF-β) that stimulate angiogenesis and matrix synthesis, leukocytes providing immunomodulation, plasma proteins facilitating scaffolding, and exosomes regulating intercellular signaling. Mechanistically, PRP enhances tissue repair through four key pathways: platelet adhesion molecules promote hemostasis and cell recruitment; immunomodulation reduces pro-inflammatory cytokines and favors M2 macrophage polarization; angiogenesis supports vascular remodeling and nutrient delivery; and serotonin-mediated pathways contribute to analgesia. These processes establish a regenerative microenvironment that supports both structural repair and functional recovery. Clinically, PRP has been applied across multiple specialties. In orthopedics, it promotes tendon, cartilage, and bone healing in conditions such as tendinopathy and osteoarthritis. In dermatology, PRP enhances skin rejuvenation, scar remodeling, and hair restoration. Gynecology has adopted PRP for ovarian rejuvenation, endometrial repair, and vulvovaginal atrophy. In dentistry and oral surgery, PRP accelerates wound closure and osseointegration, while chronic wound care benefits from its angiogenic and anti-inflammatory effects. PRP has also favored gingival recession coverage, regeneration of intrabony periodontal defects, and sinus grafting. Although preparation heterogeneity remains a challenge, PRP offers a versatile, biologically active therapy with expanding clinical utility.

## 1. Introduction

Platelet-Rich Plasma (PRP) is an autologous blood-derived product prepared through centrifugation of peripheral blood to achieve platelet concentrations exceeding physiological baseline levels. According to the Red Cross, PRP is defined as having at least 200,000 platelets per microliter (μL). This biologically active concentrate is characterized by a markedly elevated platelet density within a reduced plasma volume, typically containing two- to fivefold higher platelet counts than those found in whole blood, depending on the specific preparation protocol employed.

With the growing clinical utilization of PRP across regenerative and therapeutic disciplines, multiple classification systems have been proposed to account for variability in preparation methods, cellular composition, and intended clinical applications. Table 1 lists the classification of PRP.

The Dohan-Ehrenfest classification, proposed in 2009 [1], represents one of the earliest systematic frameworks for categorizing platelet-rich plasma (PRP) and related derivatives based on their cellular composition and fibrin architecture. This model differentiates PRP formulations by evaluating two primary parameters: (1) the presence or absence of leukocytes and (2) the structural characteristics of the fibrin network formed following platelet activation. Accordingly, the system delineates four principal PRP types: pure platelet-rich plasma (P-PRP), characterized by a platelet concentrate devoid of leukocytes and forming a low-density fibrin mesh; leukocyte- and platelet-rich plasma (L-PRP), which contains both platelets and leukocytes with a similarly loose fibrin matrix; pure platelet-rich fibrin (P-PRF), composed exclusively of platelets within a dense fibrin scaffold and lacking leukocytes; and leukocyte- and platelet-rich fibrin (L-PRF), which incorporates platelets, leukocytes, and a compact, highly cross-linked fibrin structure.

In 2012, DeLong et al. [2] proposed a more sophisticated and quantitative classification framework known as the PAW system—an acronym for Platelets, Activation, and White blood cells [2]. This model introduced standardized terminology to categorize PRP formulations based on three critical parameters: platelet concentration relative to baseline, the method of platelet activation, and the total white blood cell (WBC) content, with specific emphasis on neutrophil inclusion or exclusion. Building upon this quantitative foundation, Mautner et al. [3] in 2015 introduced the PLRA classification, which focuses on four key variables: Platelet concentration, the presence or absence of Leukocytes, Red Blood Cells (RBCs), and the method of Activation [3]. Subsequently, in 2016, Magalon et al. [4] introduced the DEPA classification system, which prioritizes the assessment of PRP preparation quality through four key dimensions—Dose, Efficiency, Purity, and Activation—thereby providing a comprehensive framework for evaluating both the composition and bioactivity of PRP products [4].

Expanding upon prior classification systems, in 2017, Lana et al. [5] underscored the regenerative contribution of peripheral blood mononuclear cells (PBMCs)—particularly monocytes and lymphocytes—owing to their capacity to secrete cytokines and growth mediators [5]. To encompass a broader range of technical and biological variables, the authors proposed the MARSPILL classification, an acronym for Method, Activation, Red blood cells, Spin, Platelets, Image guidance, Leukocytes, and Light activation. This multidimensional system provides a more comprehensive framework for characterizing PRP formulations by integrating procedural parameters with biological functionality, thereby enhancing comparability across studies.

Further refinement was achieved in 2020 with the introduction of a novel numerical classification and coding system designed to standardize PRP characterization through six quantifiable parameters [6]. This six-digit code encapsulates three principal features: platelet concentration (N1N2), cellular purity including red and white blood cell content (N3N4), and activation method (N5N6). Specifically, N1 and N2 denote the platelet concentration relative to baseline blood levels; N3 and N4 indicate the presence or absence of erythrocytes and the degree of leukocyte content; N5 and N6 specify whether the PRP is activated endogenously or exogenously, and whether calcium is added.

Platelet-rich plasma (PRP) has emerged as a multifactorial therapeutic modality that influences tissue regeneration through a diverse array of biological mechanisms. Beyond delivering concentrated growth factors, PRP exerts effects via platelet adhesion molecules, immunomodulatory mediators, angiogenic factors, and pain-regulating components.

The multifactorial mechanisms of platelet-rich plasma (PRP) underpin its broad spectrum of clinical applications across regenerative medicine. In orthopedics, PRP facilitates tendon, cartilage, and bone repair by promoting angiogenesis, modulating inflammatory cascades, and stimulating cellular proliferation, thereby demonstrating efficacy in conditions such as tendinopathy and osteoarthritis. In dermatology, PRP’s regenerative potential has been harnessed for skin rejuvenation, scar remodeling, and the treatment of androgenetic alopecia and other hair loss disorders. Gynecological applications have expanded rapidly, with PRP being utilized for endometrial regeneration, ovarian rejuvenation, and the management of vulvovaginal atrophy. In dentistry and maxillofacial surgery, PRP accelerates bone graft integration, enhances soft tissue healing, and reduces postoperative morbidity. Furthermore, in chronic wound management, PRP contributes to tissue repair through its angiogenic, anti-inflammatory, and antimicrobial properties. Collectively, these diverse applications highlight PRP’s versatility and its growing clinical relevance as a biologically active adjunct in regenerative therapies.

## 2. Methods of Literature Review

The literature has been searched to solicit basic and clinical research, which investigated the molecular and cellular mechanism of PRP preparation, classification, action and clinical utilization (applications in orthopedics, gynecology, dermatology, cosmetics and wound care). For the review, all of the articles were collected from the databases PubMed and Ovid Medline using these search terms “Platelet-rich plasma”, “platelets releasing growth factors”, “PRP” and “PDGF”. For screening and inclusion in the second stage, only English language articles with full-text were considered for inclusion in a following analysis. Duplicated articles were also excluded in this stage.

Subsequently, in order to assure the quality of retrieved studies, two experts in this field then inspected the studies to exclude articles with poorer research design, questionable methods or unclear outcomes. Eventually, a total of 230 articles were eligible for inclusion in the current review.

## 3. Preparation Methods of PRP

Currently, more than 16 commercially available autologous platelet-rich plasma (PRP) systems exist, contributing to substantial variability in collection and preparation protocols depending on the device employed [7,8]. Generally, PRP is produced through differential centrifugation, a process that separates whole blood components according to their specific gravity under controlled centrifugal force [9].

**Figure 1:**
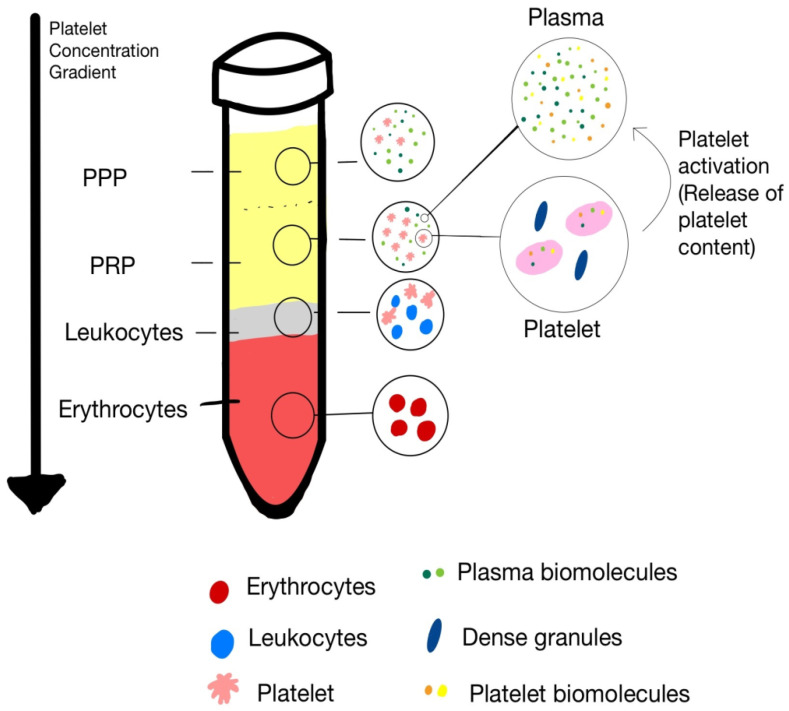
The separate blood components after differential centrifugation with respective gravity.

Two primary methods are commonly utilized for PRP preparation: the double-spin technique and the buffy coat method [10,11]. Each technique yields PRP preparations with distinct concentrations of platelets and leukocytes, and plasma constituents, allowing for customization based on the targeted clinical indication. Figure 1 illustrates the separate blood components after differential centrifugation with respective gravity.

### 3.1. Double-Spin Method [11]

The double-spin method involves two sequential centrifugation steps designed to isolate and concentrate platelets from whole blood (WB). Initially, WB is collected into tubes containing an anticoagulant solution, typically acid citrate dextrose (ACD), to prevent coagulation. It is critical that the collected blood remains at ambient temperature throughout processing, as refrigeration can compromise platelet viability and function.

The first centrifugation, often referred to as a “soft spin,” is performed at a relatively low centrifugal force. This step separates the blood into three distinct layers based on cell density: an upper plasma layer enriched with platelets and a small fraction of leukocytes; an intermediate buffy coat containing concentrated leukocytes and platelets; and a lower erythrocyte layer.

Following this initial separation, the desired fractions are collected according to the intended PRP formulation. For pure platelet-rich plasma (P-PRP), the upper plasma fraction and the superficial portion of the buffy coat are carefully aspirated and transferred to a new sterile tube. For leukocyte-rich PRP (L-PRP), the entire buffy coat along with a small portion of red blood cells may be included.

A second centrifugation step, termed a “hard spin,” is subsequently performed at higher centrifugal force to sediment the platelets into a soft pellet at the bottom of the tube. The supernatant platelet-poor plasma (PPP) is partially discarded, and the platelet pellet is resuspended in a minimal plasma volume—typically 2–5 mL—to yield the final PRP preparation.

### 3.2. Buffy Coat Method [12]

The buffy coat method represents an alternative PRP preparation technique that utilizes a single high-speed centrifugation step to isolate a concentrated layer of leukocytes and platelets. Whole blood is centrifuged at room temperature (20–24 °C), resulting in the formation of three distinct layers: a lower red blood cell fraction, a thin intermediate buffy coat containing platelets and leukocytes, and an upper layer of platelet-poor plasma (PPP).

Following centrifugation, the upper PPP layer is carefully removed, and the buffy coat is aspirated and transferred into a sterile container for further processing.

A secondary low-speed centrifugation or filtration step may be employed to further purify the platelet fraction or selectively reduce leukocyte content. This method is particularly useful when a leukocyte-rich PRP is desired, as the buffy coat inherently contains a high concentration of white blood cells.

Activation of platelets prior to application remains a debated topic [13]. While some protocols advocate the use of thrombin or calcium chloride to induce degranulation and growth factor release, recent evidence suggests that exogenous activation may not be necessary, as platelets are activated upon contact with tissue at the injection site [14].

Variations among commercial PRP systems include differences in platelet capture efficiency, required whole blood volume, centrifugation steps, and operational procedures. Selecting an appropriate system and adhering strictly to manufacturer protocols are essential to ensure consistent PRP quality and clinical efficacy.

## 4. Components of PRP

Platelet-rich plasma (PRP) is a biologically active concentrate composed of various cellular constituents—primarily platelets, leukocytes, and red blood cells (RBCs)—each contributing distinct functional roles in tissue repair and regeneration. Platelets serve as the primary effectors mediating the anabolic and regenerative actions of PRP through the release of bioactive growth factors and cytokines. In contrast, leukocytes and red blood cells exert modulatory influences that may either potentiate or impede reparative processes, contingent upon their relative concentrations, activation states, and the specific characteristics of the target tissue (Figure 1).

### 4.1. Platelets

Platelets, also referred to as thrombocytes, are anucleate cytoplasmic fragments derived from bone marrow megakaryocytes [15]. They circulate in peripheral blood at concentrations ranging from 150,000 to 450,000 platelets/μL and exhibit a lifespan of approximately 7 to 10 days. Morphologically, platelets exhibit an irregular discoid form, measuring 2–5 μm in diameter and about 0.5 μm in thickness [16]. Megakaryocytes serve as the progenitor cells of platelets, extending proplatelet processes through the endothelial barrier to release thrombocytes into the circulation [17]. Once in circulation, platelets dynamically interact with the vascular endothelium, playing a critical role in the surveillance of endothelial integrity and the early detection of vascular injury.

Platelets possess a highly specialized membrane receptor system that mediates intracellular signaling pathways essential for hemostasis [18]. Upon vascular injury, exposure of the subendothelial matrix allows von Willebrand factor (vWF) to bind to collagen, promoting platelet adhesion at the site of damage. Subsequent activation triggers platelet aggregation and interaction with fibrin, injured tissue, and neighboring platelets to form a hemostatic plug, thereby preserving vascular stability and facilitating tissue repair [19].

Platelet-rich plasma (PRP) preparations typically exhibit a 3- to 5-fold increase in growth factor (GF) concentrations relative to baseline levels, primarily due to enhanced platelet concentration and activation [20]. This enrichment enhances the localized release of bioactive mediators such as platelet-derived growth factor (PDGF), transforming growth factor-β (TGF-β), and vascular endothelial growth factor (VEGF), which are sequestered within platelet α-granules and play pivotal roles in angiogenesis, tissue regeneration, and cellular proliferation [7,21,22]. Nonetheless, supra-physiological concentrations of these factors may paradoxically attenuate regenerative efficacy, emphasizing the need for optimized PRP formulations [23].

Beyond their hemostatic function, platelets have bioactive molecules within distinct secretory granules, including α-granules, dense granules (δ-granules), and lysosome-like λ-granules [24]. Table 2 lists the key components and main functions of these granules and exosomes. The contents of these granules are either synthesized by the parent megakaryocytes during thrombopoiesis or acquired by circulating platelets via endocytic mechanisms. Collectively, these molecules modulate essential regenerative processes, including chemotaxis, cellular proliferation, differentiation, and matrix synthesis.

#### 4.1.1. α-Granules

α-granules are membrane-bound microvesicles ranging from 300 to 500 nm in diameter, and represent the most abundant type of platelet granules, accounting for roughly 10% of the total platelet volume and numbering about 50–60 per platelet [25]. These granules possess a complex proteomic composition, encompassing approximately 284 distinct proteins that participate in hemostasis, inflammation, angiogenesis, and tissue regeneration. Functionally, α-granules serve as the principal storage sites of bioactive molecules pivotal for wound healing and tissue repair. Their cargo includes adhesive proteins such as fibrinogen and von Willebrand factor; membrane glycoproteins and receptors; coagulation factors including V, XI, XIII, and prothrombin; as well as fibrinolytic proteins such as plasmin, plasminogen, and antithrombin. Moreover, α-granules contain an array of growth factors (GFs) that regulate cellular proliferation and tissue remodeling, notably platelet-derived growth factor (PDGF), transforming growth factor-β1 (TGF-β1), vascular endothelial growth factor (VEGF), basic fibroblast growth factor (bFGF), and epidermal growth factor (EGF). An overview of the components of α-granules is shown in Table 3.

#### 4.1.2. Platelet-Derived Exosomes

Upon activation, platelet-rich plasma (PRP) promotes the release of both platelet-derived granule contents and extracellular vesicles (EVs), including exosomes that originate from intracytoplasmic multivesicular bodies (MVBs) [26,27]. Exosomes are nanosized vesicles, typically measuring 30–150 nm in diameter, and are secreted by various metabolically active cell types, such as immune cells and mesenchymal stem cells (MSCs). These vesicles are widely distributed in biological fluids, including blood, bone marrow, synovial fluid, and urine [28,29,30,31]. Functionally, exosomes act as critical mediators of intercellular communication by transporting a diverse repertoire of bioactive molecules, including proteins, messenger RNAs (mRNAs), microRNAs (miRNAs), and lipids [32]. Through these molecular cargos, exosomes exert regulatory influences on immune modulation, tissue regeneration, and cellular homeostasis [33].

Platelet-derived exosomes (PLT-Exos), localized within α-granules and multivesicular bodies MVBs of platelets, constitute a major subpopulation of circulating plasma exosomes, accounting for approximately 75% of the total exosomal content [34]. Their concentration in plasma has been reported to range between 0.9 and 1.3 × 10^9^ particles/mL [35]. In 2014, Torreggiani et al. isolated exosomes from platelet-rich plasma (PRP-Exos) and showed that exosomes promote proliferation and osteogenic differentiation of bone marrow MSCs. PRP-Exos are proposed to function as nanoscale delivery vehicles, facilitating the therapeutic effects of PRP in tissue repair [36]. Importantly, platelet-derived growth factors (PGFs)—including platelet-derived growth factor (PDGF), vascular endothelial growth factor (VEGF), and transforming growth factor-β1 (TGF-β1)—can be encapsulated at high concentrations within exosomes. These bioactive molecules are subsequently transported across the extracellular matrix (ECM) to sites of tissue injury, where they promote essential reparative processes such as angiogenesis, fibroblast activation, and extracellular matrix remodeling [37,38,39].

#### 4.1.3. Dense Granules (δ-Granules)

Dense granules (δ-granules), measuring approximately 250–300 nm, are specialized platelet organelles primarily responsible for mediating hemostatic responses. These granules store small bioactive molecules such as histamine, ADP, polyphosphates, serotonin (5-hydroxytryptamine, 5-HT), and epinephrine [40]. Upon platelet activation, δ-granules release their contents to potentiate platelet aggregation, vasoconstriction, and thrombus stabilization, thereby coordinating with α-granules to sustain hemostatic function. Beyond their classical role in coagulation, several constituents of dense granules have been shown to exert immunomodulatory effects [41]. Notably, platelet-derived ADP modulates dendritic cell activity by enhancing antigen uptake and facilitating crosstalk between the innate and adaptive immune systems [42]. Furthermore, serotonin (5-HT), one of the principal mediators stored in dense granules, exhibits pleiotropic biological functions that extend beyond its established neuromodulatory role. It contributes to the regulation of nociception, inflammatory signaling, and immune cell trafficking, underscoring the multifaceted contribution of δ-granules to hemostasis and immune regulation [43,44].

#### 4.1.4. Lysosomes

The functional significance of platelet-derived lysosomes remains only partially elucidated, with limited in vivo data describing their mechanisms of release and biological activity. These organelles contain a repertoire of acid hydrolases capable of degrading glycoproteins and glycosaminoglycans, thereby contributing to intracellular catabolism and the removal of senescent or damaged cytoplasmic constituents [45]. Beyond their intracellular roles, lysosomal enzymes are also implicated in extracellular processes, including fibrinolysis, vascular remodeling, and ECM turnover [46]. Emerging evidence suggests that lysosomal proteases participate in tendon matrix homeostasis by cleaving structural components of the ECM, thereby facilitating controlled remodeling and repair [47]. Furthermore, the presence of antimicrobial proteases and cationic peptides within platelet lysosomes indicates a potential cooperative function with macrophages in innate immune defense and phagocytic clearance [48].

**Figure 2:**
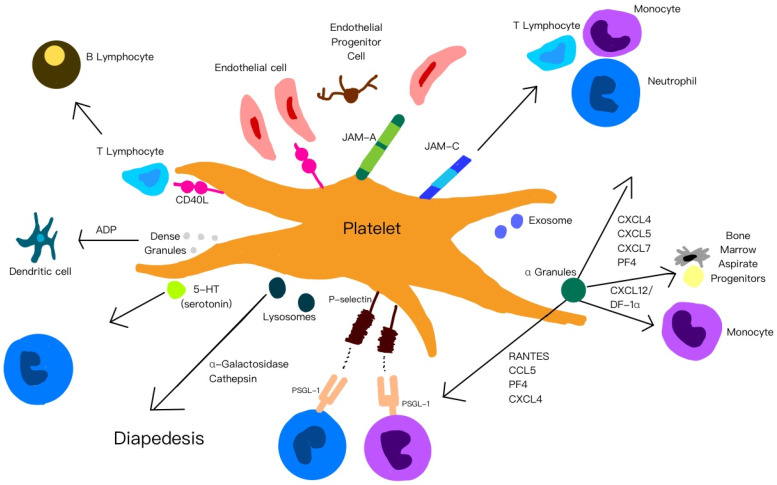
A summary of the functions of platelets, their released cytokines and chemokines, and their interactions with other cells.

Figure 2 is a summary of the functions of platelets, their released cytokines and chemokines, and their interactions with other cells.

### 4.2. Leukocytes

Leukocytes play a crucial role in modulating the inflammatory cascade, host immune defense, and the wound healing process. Among them, neutrophils are primarily responsible for initiating the inflammatory phase, while monocytes and macrophages contribute to tissue regeneration by phagocytosing necrotic tissue and cellular debris [49,50]. Furthermore, macrophages release an array of growth factors essential for tissue repair and have been implicated in the regeneration of subchondral bone.

#### 4.2.1. Neutrophils

Neutrophils are important leukocytes involved in many healing pathways, particularly through forming dense barriers against invading pathogens [51]. Beyond their traditional antimicrobial functions, recent evidence indicates that neutrophils also participate in angiogenesis and tissue regeneration through the secretion of growth and remodeling factors [52]. However, neutrophil activity can exert deleterious effects under certain conditions, as these cells release pro-inflammatory cytokines and matrix metalloproteinases (MMPs) that drive catabolic pathways and tissue degradation [53].

Azurophilic (primary) granules, which are densely packed within neutrophils, contain a wide spectrum of cytotoxic and proteolytic molecules fundamental to innate immunity. Upon activation, these granules undergo exocytosis, releasing peroxidases, lysozymes, acid hydrolases, cationic antimicrobial peptides, and serine proteases—including elastase, cathepsin G, and proteinase 3 [54,55]. These enzymes collectively mediate microbial killing and extracellular matrix degradation, thereby supporting host defense and tissue remodeling [56]. Nevertheless, excessive proteolytic activity can inadvertently damage surrounding healthy tissues, as demonstrated in experimental models of tendon degradation [22].

Specific (secondary) granules of neutrophils are characterized by their larger size and lower abundance relative to azurophilic (primary) granules. While there is partial overlap in granule content—such as lactoferrin, gelatinase, and lysozyme—secondary granules are also enriched with enzymes that contribute to ECM remodeling [57]. These include matrix metalloproteinases, particularly MMP-8 (neutrophil collagenase) and MMP-9 (gelatinase B), which degrade fibrillar and basement membrane components [58]. In addition, secondary granules contain a range of antimicrobial peptides such as cathelicidins, and host defense proteins like secretory leukocyte protease inhibitor (SLPI) [55]. Lactoferrin, a key constituent, exhibits broad-spectrum antimicrobial activity by chelating iron and directly inhibiting bacterial, viral, fungal, and parasitic pathogens [59]. Its antiviral properties are attributed to its competitive interaction with glycosaminoglycans, thereby impeding viral adherence and cellular entry—an action that may also impact cartilage proteoglycans or synovial hyaluronan structures.

Tertiary granules, relatively sparse and diminutive, release MMP-9, MMP-15, and metal-binding peptides such as NGAL and NRAMP1, which contribute to extracellular matrix remodeling and antimicrobial defense by restricting metal availability to engulfed pathogens [56].

#### 4.2.2. Monocytes

The monocyte content within platelet-rich plasma (PRP) preparations varies depending on the isolation and processing method; however, their functional contributions to tissue regeneration remain insufficiently characterized. Derived from hematopoietic stem cells, monocytes circulate in peripheral blood and migrate toward injured or inflamed tissues in response to local chemotactic signals, where they differentiate into macrophages (M) or dendritic cells, forming integral components of the mononuclear phagocyte system (MPS) [60]. Upon tissue injury, monocytes exhibit remarkable plasticity, differentiating into distinct macrophage phenotypes according to the microenvironmental milieu [60]. Among these macrophages, classically activated M1 secrete pro-inflammatory cytokines (e.g., IFN-γ), nitric oxide, VEGF, and FGF, supporting microbial defense and angiogenesis. Alternatively activated M2, which includes subtypes such as M2a, M2b, and M2c, exhibits anti-inflammatory and reparative functions, producing IL-10, ECM components, and angiogenic factors [61]. Importantly, in vivo evidence indicates that macrophages can undergo dynamic phenotype switching from M1 to M2 states, a process regulated by cytokine signaling, notably by interleukin-4 (IL-4) [62].

#### 4.2.3. Lymphocytes

Mononuclear T and B lymphocytes are typically enriched relative to other leukocyte populations. These cells are fundamental components of the adaptive immune system, mediating antigen-specific cytotoxic responses and immunological memory [63]. T lymphocytes, through the secretion of cytokines such as interferon-γ (IFN-γ) and interleukin-4 (IL-4), exert a regulatory influence on macrophage polarization, thereby shaping the local immune reaction [62].

### 4.3. Red Blood Cells

RBCs are largely excluded during platelet-rich plasma (PRP) preparation because of their limited regenerative capacity and potential cytotoxic effects. While RBCs play an essential physiological role in oxygen and nutrient transport through hemoglobin, their degradation—particularly under oxidative stress—can release free iron and generate reactive oxygen species (ROS), which induce apoptosis and tissue damage [8,64]. Consequently, minimizing or eliminating RBC contamination in PRP formulations intended for intra-articular applications is critical to prevent inflammatory and degenerative sequelae.

### 4.4. Plasma

Plasma, the fluid matrix of blood, serves as a dynamic reservoir of biomolecules, reflecting systemic physiological and pathological states. While core plasma proteins—such as albumin, immunoglobulins, complement, and coagulation factors—are well characterized, proteomic studies have identified over 1500 proteins [65]. Broadly, plasma proteins can be categorized into three main groups: (1) highly abundant native proteins, (2) tissue-derived “leakage” proteins indicative of pathological states [65], and (3) regulatory cytokines implicated in immune and inflammatory responses [66]. The total plasma protein concentration ranges from 60 to 80 mg/mL, with albumin comprising approximately 60%, globulins about 35%, and fibrinogen around 4%. The remaining ~1% encompasses thousands of low-abundance proteins, many of which serve essential functions in intercellular signaling, homeostatic regulation, and disease pathogenesis [67,68].

Targeted utilization of enriched plasma proteins has emerged as a promising therapeutic strategy in regenerative medicine and tissue engineering, particularly for enhancing tissue repair and musculoskeletal regeneration. Structural plasma proteins such as fibronectin, fibrin, and vitronectin not only facilitate hemostasis but also promote stromal cell adhesion and migration, thereby accelerating tissue restoration [69]. In addition, plasma serves as a carrier for a diverse array of circulating hormones, including thyroxine, adrenocorticotropic hormone, androgens, estrogens, progesterone, and human growth hormone. Among these, insulin-like growth factor-1 (IGF-1) has been identified as a pivotal mediator of musculoskeletal repair, with accumulating evidence demonstrating its efficacy in enhancing tendon and cartilage healing in experimental models [70,71]. In contrast, the specific roles of other plasma-derived hormones in platelet regulation and musculoskeletal metabolism remain incompletely defined. Glucocorticoids generally exert protective effects by suppressing inflammatory mediator activity and mitigating tissue damage. Hormonal regulation of platelet behavior is also evident: testosterone promotes intracellular epidermal growth factor synthesis, while estrogens inhibit it. Moreover, physiological concentrations of dihydrotestosterone (DHT) have been shown to attenuate platelet aggregation and reduce thromboxane A_2_ release in PRP preparations [72]. Although estrogens alone exert minimal influence on platelet aggregation, they may synergize with adenosine diphosphate (ADP) or epinephrine to amplify the aggregatory response [73].

## 5. Tissue Repair and Regeneration Provoked by PRP

### 5.1. Platelet Adhesion Molecules

To elucidate the mechanisms underlying platelet-rich plasma (PRP) activity in inflamed tissues and the immunomodulatory roles of platelets, it is crucial to examine platelet surface receptors—particularly integrins and junctional adhesion molecules (JAMs)—and their involvement in mediating cellular interactions central to both innate and adaptive immunity. Integrins are transmembrane adhesion receptors ubiquitously expressed across diverse cell types and are highly abundant on platelets. The major platelet integrins include α5β1, α6β1, α2β1 (LFA-2 or GPIa/IIa), and αIIbβ3 (GPIIb/IIIa) [74]. In their resting state, these integrins exhibit low ligand-binding affinity; however, upon platelet activation, conformational rearrangements markedly enhance their binding affinity and specificity. Each integrin subtype performs distinct yet complementary roles, facilitating platelet adhesion to endothelial cells, leukocytes, and components of the extracellular matrix [75]. In addition, the GPIb–V–IX complex on platelets binds to von Willebrand factor (vWF), enabling platelet adhesion to exposed subendothelial surfaces during vascular injury and initiating hemostasis [76]. In concert with integrins, these adhesion factors contribute to inflammatory signaling cascades. In particular, integrin αIIbβ3 stabilizes platelet–leukocyte complexes by bridging to neutrophil Mac-1 receptors through fibrinogen, thereby promoting leukocyte recruitment and activation [77].

Platelets, neutrophils, and vascular endothelial cells express selectins, a family of cell adhesion molecules involved in mediating cellular interactions during inflammation [78]. Activated platelets express P-selectin, which binds to PSGL-1 on neutrophils and monocytes [79], triggering signaling that activates neutrophil integrins Mac-1 and LFA-1. Mac-1 then binds to platelet GPIb or GPIIb/IIIa via fibrinogen, while LFA-1 binds to intercellular adhesion molecule-2 (ICAM-2) on platelets, both interactions reinforcing stable neutrophil–platelet adhesion [80]. Additionally, LFA-1 can bind to ICAM-2 on platelets, further reinforcing the adhesion and promoting sustained cellular interactions [81].

### 5.2. Anti-Inflammatory Effects

Platelet activation during acute or chronic inflammatory conditions triggers the release of growth factors, cytokines, chemokines, and other signaling molecules that attract neutrophils and monocytes [82]. Djenek et al. demonstrated that platelet–leukocyte interactions within leukocyte-rich PRP (LR-PRP) attenuate inflammatory responses by downregulating key cytokines, including interleukin-1 (IL-1), interleukin-6 (IL-6), and tumor necrosis factor-α (TNF-α) [83]. In addition, platelet activation facilitates monocyte chemotaxis toward inflamed tissues, where these cells differentiate into anti-inflammatory M2 macrophages. This process enhances the release of IL-4 and IL-10 [84,85] while concurrently suppressing TNF-α-driven chemotaxis and proteolytic activity [86]. Furthermore, activated platelets contribute to the activation of regulatory T cells (Tregs) and secrete a variety of chemokines—including platelet factor 4 (PF-4), regulated on activation, normal T cell expressed and secreted (RANTES), interleukin-1 (IL-1), and C-X-C motif chemokine ligand 12 (CXCL-12)—which collectively prevent monocyte apoptosis and promote macrophage differentiation [87]. Importantly, platelet-derived factors also suppress nuclear factor kappa B (NF-κB) activation, thereby reducing pro-inflammatory signaling, particularly in synovial cells [88].

### 5.3. Immunomodulation

Innate and adaptive immune systems work synergistically to protect the host against infection, with monocytes, macrophages, neutrophils, and natural killer (NK) cells constituting the primary mediators of innate immunity, whereas lymphocytes orchestrate adaptive immune responses [89]. The innate immune system provides a rapid, non-specific defense through pattern recognition receptors (PRRs), such as Toll-like receptors (TLRs) and RIG-I-like receptors (RLRs), which activate nuclear factor kappa B (NF-κB) signaling pathways [90]. Platelets, acting as early responders to vascular injury and microbial invasion, express PRRs and immunomodulatory molecules—including P-selectin, CD40 ligand (CD40L), and interleukin-1β (IL-1β)—that facilitate interactions with leukocytes [91]. These interactions promote neutrophil recruitment, oxidative bursts generation [92], neutrophil extracellular trap (NET) formation [93], and monocyte activation, while also influencing dendritic cell (DC) recruitment and maturation, thereby bridging innate and adaptive immune responses [75].

On the other hand, the adaptive immune system—driven by antigen-specific T and B lymphocytes—mounts a slower but highly targeted defense that confers immunological memory. Helper T (Th) cells (Th1, Th2, Th17) and cytotoxic T (Tc) cells coordinate specialized effector functions, while Th2 cells and IL-4 promote anti-inflammatory M2 macrophage polarization and tissue repair [94]. Platelets contribute to adaptive immunity by expressing CD40L [95], enhancing T-cell activation and supporting B-cell isotype switching and proliferation. Thus, platelet-leukocyte interactions are integral to both immune defense and regeneration, highlighting the roles of platelets as key modulators of immune function across both systems.

**Figure 3:**
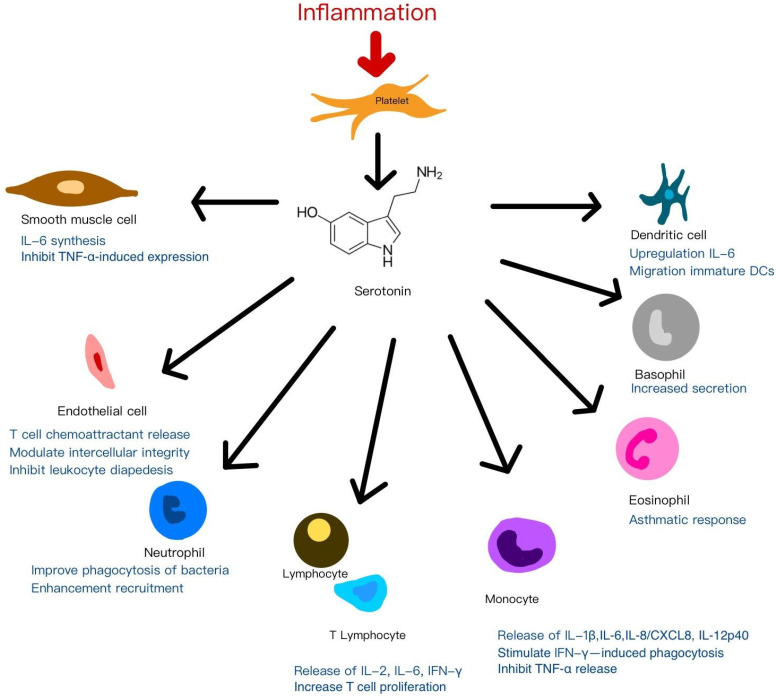
The mechanisms of anti-inflammatory and immunomodulation effects of platelets by serotonin signaling to adjacent cells.

Beyond these established roles, emerging evidence highlights serotonin (5-hydroxytryptamine, 5-HT)—particularly platelet-derived 5-HT—as an additional immunomodulatory mediator acting through various serotonin receptors (5-HTRs) expressed on immune cells. Depending on the specific receptor profile, 5-HT modulates both innate and adaptive immune responses [96]. Platelet-derived 5-HT has been shown to enhance regulatory T-cell (Treg) proliferation and modulate B-cell, NK-cell, and neutrophil activity, partly by promoting dendritic cell and monocyte recruitment to inflammatory sites [97,98]. Within inflamed microenvironments, this serotonergic signaling by PRP may influence immune cell dynamics, inflammatory progression, and clinical outcomes in regenerative applications. Figure 3 illustrates the mechanisms of anti-inflammatory and immunomodulation effects of platelets by serotonin signaling to adjacent cells.

### 5.4. Angiogenesis

Angiogenesis, a fundamental process in tissue regeneration, is tightly regulated by the dynamic balance between pro-angiogenic mediators—such as vascular endothelial growth factor (VEGF)—and anti-angiogenic molecules, including angiostatin and thrombospondin-1 (TSP-1). Platelet-rich plasma (PRP) contains both pro-angiogenic factors (e.g., VEGF, transforming growth factor-β [TGF-β], basic fibroblast growth factor [bFGF]) and anti-angiogenic mediators (e.g., platelet factor 4 [PF4], TSP-1), whose net biological effects depend on platelet activation status and receptor-mediated signaling. Although PRP exhibits both stimulatory and inhibitory influences on angiogenesis, its overall effect is predominantly pro-angiogenic, particularly in therapeutic contexts such as wound repair and tissue regeneration. Mechanistically, PRP enhances endothelial cell proliferation, capillary sprouting, and neovascularization through the coordinated action of key growth factors—especially VEGF and platelet-derived growth factor-BB (PDGF-BB)—whose synergistic interaction has been shown to promote vascular network formation more effectively than either factor alone [99]. Additionally, PRP stimulates vasculogenesis and arteriogenesis via stromal cell-derived factor-1α (SDF-1α) and its receptor CXCR4 in endothelial progenitor cells, thereby supporting vascular remodeling and tissue perfusion [100]. Importantly, optimal angiogenic responses are concentration-dependent, with 1.5 × 10^6^ platelets/μL identified as the most effective threshold for promoting vascularization [101]. Conversely, excessively high platelet concentrations may paradoxically inhibit angiogenesis [102], underscoring the importance of appropriate PRP preparation and dosing strategies.

### 5.5. Analgesic Effects

Platelet-rich plasma (PRP) releases a spectrum of bioactive mediators that modulate inflammation and nociception, thereby altering the local tissue microenvironment to facilitate repair. Despite incomplete mechanistic understanding, PRP has been widely applied in the management of chronic pain disorders, particularly orthopedic and spinal pathologies. Everts et al. [103] conducted the first randomized controlled trial (RCT) demonstrating that PRP derived from autologous buffy coat and activated with thrombin significantly reduced postoperative pain, decreased opioid consumption, and improved postoperative rehabilitation following shoulder surgery. These effects were attributed, at least in part, to platelet-derived serotonin (5-hydroxytryptamine, 5-HT) released upon activation. Platelet activation induces the exocytosis of intracellular α- and dense granules rich in 5-HT [104], a neurotransmitter known to play a pivotal role in pain modulation. Clinical PRP (C-PRP), characterized by a 5–7-fold increase in platelet count, may result in enhanced 5-HT release and augmented analgesic effects.

Peripheral serotonin is predominantly stored in platelets and, to a lesser extent, in mast cells and endothelial cells. Upon tissue injury or platelet activation, 5-HT is released into the extracellular milieu, where it modulates nociceptive signaling through multiple receptor subtypes, including 5-HT_1_, 5-HT_2_, 5-HT_3_, 5-HT_4_, and 5-HT_7_ [105,106]. These receptors are expressed across sensory neurons and immune cells, and can either promote or inhibit pain transmission depending on the receptor subtype and tissue context. In chronic pain conditions, dysregulation of peripheral 5-HT signaling has been observed, providing the rationale for the therapeutic use of serotonin-modulating agents such as selective serotonin reuptake inhibitors (SSRIs) and serotonin-norepinephrine reuptake inhibitors (SNRIs) [107]. Although PRP is recognized as a reservoir of platelet-derived 5-HT, the precise contribution of this mediator to its overall analgesic effect remains unclear. Further mechanistic studies are warranted to elucidate the role of PRP-derived 5-HT in pain modulation, particularly within chronically inflamed or degenerative tissues.

Preclinical investigations in animal models have explored the analgesic efficacy of PRP, though methodological variability has limited cross-study comparisons [108]. Clinical findings are likewise heterogeneous: some studies reported minimal analgesic benefit in tendinopathies and rotator cuff injuries [109,110], whereas others demonstrated significant pain reduction in conditions such as tendinosis, osteoarthritis, plantar fasciitis, and foot and ankle disorders [111,112]. Notably, Kuffler demonstrated that PRP reduced or eliminated chronic neuropathic pain in patients with non-regenerated nerve injuries, with effects lasting up to six years and onset of relief within three weeks post-treatment [113].

Recent studies have reported analgesic effects of PRP in post-surgical wound care, highlighting the role of vascular injury, hypoxia, and the benefits of neoangiogenesis in pain reduction. Additionally, a systematic review and meta-analysis by Johal et al. confirmed PRP’s pain-relieving effects in orthopedic conditions, especially lateral epicondylitis and knee osteoarthritis [114]. However, critical parameters such as leukocyte content, platelet concentration, and activation methods were not detailed. In a rat tendinopathy model, full pain relief was achieved with a platelet concentration of 1.0 × 10^6^/μL, while lower concentrations were less effective [108]. Further studies are needed to determine the optimal PRP composition for analgesia.

## 6. The Applications of PRP

### 6.1. Wound Healing

Wound healing is a highly orchestrated biological process encompassing sequential yet overlapping phases of inflammation, tissue formation, and remodeling, all of which involve coordinated cell proliferation, migration, angiogenesis, and extracellular matrix (ECM) deposition [115]. Disruption of these tightly regulated mechanisms can lead to chronic non-healing wounds—commonly resulting from trauma, venous insufficiency, pressure injury, diabetes mellitus, or arterial occlusion—which substantially impair patient quality of life and impose significant socioeconomic and healthcare burdens [116]. Standard treatments such as debridement and dressing changes often yield suboptimal outcomes, highlighting the need for novel regenerative therapies.

PRP has emerged as a promising, minimally invasive, and cost-effective approach for wound healing, offering an alternative to conventional therapies. Its clinical utility was first demonstrated in the management of chronic leg ulcers, where PRP facilitated the formation of vascularized granulation tissue and accelerated re-epithelialization [117]. Guo et al. conducted the first study to elucidate the role of the Erk and Akt signaling pathways in PRP, identifying them as key regulators of exosome biogenesis [27]. These exosomes were shown to enhance cell proliferation, migration, and angiogenesis, contributing to tissue regeneration.

In chronic conditions like diabetic ulcers, PRP promotes redox homeostasis by balancing reactive oxygen species (ROS) and inflammatory mediators, thereby expediting tissue repair [118]. Clinical studies consistently report significant reductions in wound size, pain, and inflammation, with minimal adverse effects. For example, in a cohort of 150 patients with diabetic foot ulcers, topical PRP application markedly enhanced granulation tissue formation and accelerated wound closure [119], while other investigations demonstrated improved neovascularization and healing outcomes even in immunocompromised populations [120].

PRP has also been effective in second intention healing, where wound closure depends on granulation and epithelization rather than primary closure. Studies in animal models—including canine and rabbit wound models—have shown that intralesional PRP administration enhances tissue perfusion, angiogenesis, granulation tissue maturation, and collagen synthesis, collectively accelerating the healing process [121]. Moreover, PRP exhibits synergistic effects when combined with bone marrow-derived mesenchymal stem cells (BM-MSCs), augmenting cell proliferation and differentiation within a regenerative microenvironment [122]. Collectively, these findings substantiate the therapeutic potential of PRP as a biologically active adjunct for the management of both acute and chronic wounds.

### 6.2. The Application of PRP in Gynecology

PRP usage is still emerging in the field of gynecology, with most use in reproductive medicine and some in wound healing and urinary tract conditions.

#### 6.2.1. Ovary

Intraovarian platelet-rich plasma (PRP) therapy has been investigated as a potential treatment for managing ovarian dysfunction and diminished ovarian reserve. Studies have demonstrated increases in retrieved oocyte numbers [123], elevated anti-Mullerian hormone (AMH) levels, reduced follicle-stimulating hormone (FSH) concentrations, and trends toward improved clinical pregnancy and live birth rates in women with premature ovarian insufficiency [124,125,126]. One proposed mechanism for PRP-induced fertility restoration is the activation of dormant oocytes in response to PRP-derived growth factors. Alternatively, PRP may enhance the ovarian microenvironment, promoting differentiation of pluripotent ovarian stem cells into germ cells—a hypothesis supported by evidence of de novo oogenesis from resident ovarian germline stem cells [127,128]. Ovarian folliculogenesis progresses through two major phases: an initial preantral, gonadotropin-independent stage governed by intraovarian paracrine signaling, and a subsequent gonadotropin-dependent stage. The preantral phase is regulated by autocrine and paracrine factors such as bone morphogenetic proteins (BMPs), growth differentiation factors (GDFs), transforming growth factor-β (TGF-β), activins, inhibins, and AMH. Among these, growth differentiation factor 9 (GDF9), secreted by oocytes, is essential for cumulus cell expansion and follicular maturation [129]. Collectively, these findings reinforce the concept of the ovary as a direct target for PRP-based therapies, with PRP potentially promoting folliculogenesis and neoangiogenesis via its rich concentration of bioactive growth factors.

Autologous intraovarian PRP has emerged as a potential strategy to counteract age-related reproductive decline. In a case series of three menopausal women aged 27, 40, and 46, PRP injections (4 mL per ovary, ~250,000/µL platelets) led to resumed menstrual cycles within 1–1.2 months and natural conception within 2–6 months, resulting in ongoing pregnancies beyond the second trimester [130]. However, variability in ovarian response and procedural challenges, such as PRP leakage from atrophic ovaries, highlight the need for standardization and further investigation.

In a murine model, co-administration of PRP with bone marrow-derived stem cells showed synergistic effects, including improved follicular growth, reduced oocyte fragmentation, better chromosomal alignment, and enhanced blastocyst formation. These benefits were accompanied by improved mitochondrial function and decreased oxidative stress, suggesting a multifaceted mechanism of ovarian rejuvenation [131].

#### 6.2.2. Endometrium

PRP has been investigated as a novel therapeutic option to enhance endometrial thickness and implantation outcomes, particularly in patients with thin or refractory endometrium. In one study, intrauterine PRP infusion significantly increased endometrial thickness (>9.0 mm) in all 19 IVF patients, with a 73.7% positive pregnancy rate and 26.3% live birth rate [132]. However, a randomized study by Tehraninejad et al. [133] reported no significant improvement in implantation or pregnancy rates among patients with normal endometrial thickness and repeated implantation failure (RIF) following PRP treatment. These findings suggest that the therapeutic effect of PRP may be population-dependent, with greater benefit observed in cases of refractory endometrium.

Chronic endometritis (CE) represents a persistent inflammatory condition of the endometrial lining, typically diagnosed via hysteroscopic biopsy demonstrating plasma cell infiltration [134]. Elevated levels of proinflammatory cytokines such as IL-6, IL-1β, and TNF-α are characteristic of CE and are known to impair endometrial cell migration and proliferation [135]. As CE is frequently associated with recurrent implantation failure and pregnancy loss, PRP has emerged as a promising autologous therapy. A case report demonstrated complete resolution of CE and a successful live birth following intrauterine PRP infusion in a patient with six failed embryo transfers [136]. In vitro bovine studies further revealed that PRP downregulates IL-1β, IL-8, and iNOS, while upregulating ER-α, ER-β, and PR—genes critical for endometrial receptivity and pregnancy maintenance [137]. Furthermore, PRP increases the expression of MMP3, MMP7, and MMP26 in endometrial stromal and stem cells, which are essential for tissue remodeling and healing [138,139].

PRP has also been explored in the management of Asherman’s syndrome, a condition characterized by basal endometrial damage, intrauterine adhesions, and impaired endometrial regeneration, often resulting in infertility, abnormal menstruation, and pregnancy loss [140,141]. Aghajanova et al. reported two successful pregnancies post-PRP following adhesiolysis, suggesting improved endometrial function even in cases without measurable thickness gain [141]. Similarly, a study using a higher PRP volume (5 mL subendometrial injection plus 5 mL PRP gel coating) in 30 women showed reduced adhesion reformation and significantly prolonged menstruation duration [142].

Overall, while PRP shows promising results in endometrium thickness and pregnancy outcomes, its effectiveness remains variable depending on the patient population and clinical context.

#### 6.2.3. Urogynecology

PRP has shown positive effects in treating various urogynecological disorders. In patients with recurrent vesicovaginal fistulas, PRP injection around the fistulous tract prior to Latzko repair resulted in complete healing without scarring or urinary dysfunction in all treated cases [143]. In cystocele repair, adjunctive use of PRP significantly reduced recurrence rates and alleviated prolapse symptoms compared with surgery alone [144]. Likewise, Gorlero et al. reported 80% anatomical success and 100% symptom improvement in pelvic organ prolapse surgery enhanced with platelet-rich fibrin, though the study was limited by a small sample size [145].

Stress urinary incontinence (SUI) is a prevalent condition that markedly impairs quality of life and is primarily attributed to defects of the pubourethral ligament (PUL), as proposed by the integral theory. The PUL provides structural support to the bladder neck and midurethra, and its disruption has been associated with persistent SUI in animal models. PRP contains multiple growth factors—such as VEGF, IGF-I, PDGF, HGF, TGF-β, and FGF—that may facilitate ligament repair and tissue regeneration, providing a mechanistic basis for its potential use in SUI treatment. In a pilot study, periurethral PRP injection achieved a 60% success rate, indicating potential benefit in selected patients [146].

PRP has also been applied in interstitial cystitis/painful bladder syndrome (IC/PBS). In a rat model of cyclophosphamide-induced cystitis, intravesical PRP reduced inflammation and improved bladder barrier function [147]. When combined with hyaluronic acid, PRP treatment prolonged voiding intervals, increased tight-junction protein (ZO-2) expression, and reduced IL-6 levels, reflecting enhanced urothelial repair and reduced inflammation. Building on these findings, Jhang et al. [148] conducted a clinical study in 19 IC/BPS patients receiving four monthly PRP bladder instillations. The treatment led to improvements in bladder capacity, urinary flow rate, pain, and symptom scores. Histologic analysis further revealed upregulation of ZO-1, E-cadherin, and TGF-β, supporting PRP’s role in restoring urothelial barrier function and attenuating inflammation. In addition, a proteomic study in women with overactive bladder (OAB) identified vascular cell adhesion molecule-1 (VCAM-1) as the only urinary protein significantly different from controls, suggesting a possible role as a diagnostic biomarker that warrants validation in larger cohorts [149]. Collectively, current evidence supports PRP as a potentially safe and effective adjunct therapy for various urogynecological disorders, including vesicovaginal fistula, pelvic organ prolapse, SUI, and IC/BPS.

However, given the limited sample sizes and methodological variability among available studies, larger randomized controlled trials are warranted to validate these preliminary findings and establish standardized treatment protocols.

#### 6.2.4. Vaginal Atrophy

Vaginal atrophy in postmenopausal women often leads to local dryness, itching, and volume loss. The combination of PRP with other treatments—such as autologous lipofilling, hyaluronic acid, and photodynamic therapy—has shown encouraging outcomes in managing genitourinary syndrome of menopause [150].

### 6.3. The Application of PRP in Dermatology

#### 6.3.1. Hair Disorders

Platelet-rich plasma (PRP) therapy has been extensively investigated as a therapeutic modality for non-scarring alopecias, particularly androgenetic alopecia (AGA), androgenic alopecia, alopecia areata (AA), and lichen planopilaris [151,152,153,154,155,156,157,158]. While androgenetic alopecia and androgenic alopecia are often used interchangeably and both alopecias are linked to the effects of androgens (such as dihydrotestosterone; DHT) on hair follicles, these two terms belong to different entities. Androgenetic alopecia is a genetic disorder leading to pattern hair loss (male/female pattern baldness), while androgenic alopecia is a broader term for hair loss caused by excess androgens, such as those from a tumor or supplement. The two are often misused, but androgenetic alopecia has a normal or even low level of androgens; the hair follicles are simply more sensitive to them due to genetic factors.

As mentioned above, androgenetic alopecia (AGA) is a hereditary, androgen-dependent patterned hair loss that occurs despite normal systemic androgen levels, whereas androgenic alopecia denotes non-patterned hair loss directly caused by androgen excess, either endogenous or exogenous in origin. The regenerative effects of PRP are primarily mediated through the release of multiple growth factors from activated platelets, which collectively enhance hair follicle proliferation, angiogenesis, and prolongation of the anagen phase of the hair cycle [159,160]. Mechanistically, PRP activates several key intracellular signaling cascades—most notably the extracellular signal-regulated kinase (ERK) and protein kinase B (Akt) pathways. The Akt pathway exerts anti-apoptotic effects via upregulation of BCL-2, thereby enhancing follicular cell survival, while the ERK pathway facilitates cellular proliferation and growth [157,158,161,162]. Among the numerous bioactive molecules released by PRP, vascular endothelial growth factor (VEGF) and platelet-derived growth factor (PDGF) play particularly important roles. VEGF stimulates perifollicular angiogenesis and increases microvascular permeability, whereas PDGF promotes the transcription of genes that initiate and regulate the anagen phase [160,163]. Additionally, transforming growth factor-β (TGF-β) modulates immune responses by suppressing monocyte chemoattractant protein-1 (MCP-1), thereby reducing immune cell infiltration into the follicular microenvironment. Through this cytokine-mediated regulation, TGF-β may contribute to the restoration of immune tolerance and attenuation of inflammation in alopecia areata [164,165].

Recent clinical studies have provided strong evidence supporting the therapeutic potential of PRP in androgenetic alopecia. Clinical trials have shown that PRP significantly increases hair density compared with baseline, although the improvements were not statistically different from placebo, and outcomes were comparable to those achieved with topical minoxidil [154,157]. Moreover, PRP has demonstrated benefits in female AGA and in lichen planopilaris when combined with low-dose naltrexone [155,156]. Further, standardized clinical protocols proposed by Aristizabal et al. [158] (2024) have enhanced the safety, consistency, and reproducibility of PRP application in AGA management [158].

Collectively, these mechanisms underscore the multifactorial nature of PRP’s action in hair restoration, encompassing angiogenic stimulation, follicular cell activation, and immunomodulation. Despite interindividual variability related to patient age and platelet growth factor content, a growing body of evidence from in vitro, animal, and clinical studies supports the therapeutic potential of PRP in the management of androgenetic alopecia [151,166].

#### 6.3.2. Pigmentary Disorders

The mechanisms underlying PRP’s effects in melasma and vitiligo remain incompletely understood.

##### Melasma

Platelet-rich plasma (PRP) appears to ameliorate melasma through two principal mechanisms: inhibition of melanogenesis and enhancement of dermal matrix synthesis. A key mediator in this process is transforming growth factor-β1 (TGF-β1), which suppresses PAX3 expression and consequently downregulates microphthalmia-associated transcription factor (MITF) and its downstream targets—tyrosinase, tyrosinase-related protein 1 (Tyrp1), and dopachrome tautomerase (DCT)—thereby attenuating melanin synthesis [167]. In addition, TGF-β1 inhibits extracellular signal-regulated kinase (ERK) activation, which indirectly reduces tyrosinase gene transcription, and promotes the expression of skin basement membrane components such as laminin, collagen IV, and tenascin [168]. Moreover, the anti-inflammatory effects of PRP may mitigate elevated inflammatory markers observed in melasma-affected skin, such as interleukin-17 (IL-17), CD4-positive T cells, and cyclooxygenase-2 (COX-2) [169,170].

##### Vitiligo

PRP has shown therapeutic potential in vitiligo by promoting melanocyte regeneration, enhancing intercellular adhesion, and exerting anti-inflammatory effects [165]. Growth factors contained in PRP activate the Akt and BCL-2 signaling pathways, thereby preventing melanocyte apoptosis and upregulating β-catenin signaling, which supports melanocyte proliferation and survival [171]. Moreover, PRP provides structural proteins such as fibrin, fibronectin, and vitronectin, which strengthen cell–cell and cell–matrix adhesion, counteracting the effects of fibroblast growth factor (FGF) and keratinocyte growth factor (KGF) deficiency—both implicated in melanocyte detachment and loss [172]. Additionally, PRP stimulates keratinocyte and fibroblast proliferation, enhancing their interaction with melanocytes [173]. The anti-inflammatory properties of PRP further contribute to its therapeutic action. PRP has been shown to suppress the expression of key proinflammatory cytokines, including interleukin-1 (IL-1), interferon-gamma (IFN-γ), and tumor necrosis factor-alpha (TNF-α), all of which are critically involved in the pathogenesis of vitiligo [174]. Furthermore, transforming growth factor-beta (TGF-β) enhances this immunomodulatory effect by regulating T-cell-mediated immune responses [175]. Collectively, these findings suggest that PRP may offer multifactorial therapeutic benefits in both pigmentary disorders via melanocyte regulation, immune modulation, and extracellular matrix restoration.

#### 6.3.3. Rejuvenation and Scars

PRP has gained widespread clinical use in skin rejuvenation and the management of various scar types, including acne, atrophic, hypertrophic, and keloidal scars [176,177]. In aging or photo-damaged skin, characterized by reduced elastin, fragmented fibers, and impaired collagen synthesis, PRP promotes fibroblast proliferation, enhances matrix metalloproteinase (MMP) activity, and stimulates collagen production and extracellular matrix remodeling [165]. Furthermore, PRP upregulates intracellular antioxidant enzymes, thereby mitigating UV-induced oxidative stress and improving overall dermal resilience [178].

Fibroblasts play a pivotal role in skin regeneration and scar remodeling through their interactions with keratinocytes and their production of extracellular matrix components [165]. PRP has been shown to promote fibroblast proliferation in a dose-dependent manner [179], mainly via activation of transforming growth factor-beta (TGF-β) and fibroblast growth factor (FGF) signaling [180]. TGF-β1, in particular, enhances type I collagen synthesis while inhibiting its degradation [181], whereas increased expression of MMP-1 and MMP-2 facilitates matrix remodeling and scar maturation [182,183]. In hypertrophic scars, PRP helps to reestablish a balanced collagen ratio by reducing type III and increasing type I collagen [184,185]. Collectively, through modulation of fibroblast activity, collagen homeostasis, and oxidative balance, PRP supports both skin rejuvenation and functional scar remodeling, offering a biologically sound adjunct to conventional aesthetic and reconstructive therapies.

#### 6.3.4. Lichen Sclerosus and Other Inflammatory Disorders

PRP has shown promising effects in lichen sclerosus (LS), a chronic inflammatory dermatosis predominantly affecting the anogenital region [186]. PRP is proposed to promote extracellular matrix remodeling, angiogenesis, and reducing inflammation. It also counteracts oxidative stress and modulates miR-155, helping to prevent excessive scarring. Furthermore, PRP mitigates oxidative stress and modulates microRNA-155 (miR-155) expression, a key regulator implicated in LS pathogenesis. Dysregulated miR-155 expression in macrophages and T lymphocytes has been associated with impaired regulatory T-cell (Treg) function, leading to chronic inflammation, loss of immune tolerance, and excessive collagen deposition, which contribute to dermal fibrosis and sclerosis [187,188]. Additionally, the antioxidant properties of PRP may help preserve tumor suppressor gene activity, thereby potentially reducing the long-term risk of squamous cell carcinoma in LS patients [139,178].

Beyond LS, PRP demonstrates anti-inflammatory, immunomodulatory, and regenerative effects across various inflammatory dermatoses—including psoriasis, Behçet disease, morphea, inflammatory nail disorders, and oral lichen planus—through the regulation of cytokine expression, enhancement of tissue repair, and modulation of oxidative stress.

### 6.4. The Application of PRP in Orthopedics

#### 6.4.1. Bone Fracture Healing

Bone healing proceeds through three stages: an initial inflammatory phase with vascular ingrowth, a repair phase involving fibroblast migration, collagen deposition, and soft callus formation, and a final remodeling phase where the bone regains its original structure and strength [178]. Adequate vascularization is critical throughout all stages to ensure successful regeneration and nutrient delivery [189].

The osteogenic efficacy of PRP is primarily attributed to its content of growth factors and cytokines, which modulate inflammation and promote the osteogenic differentiation of marrow-derived mesenchymal stem cells (MSCs) within the repair microenvironment [190,191]. Through these mechanisms, PRP supports early bone formation, enhances angiogenesis, and reduces the risk of delayed union or nonunion. Among its key osteoinductive constituents, bone morphogenetic protein-2 (BMP-2) plays a pivotal role in promoting MSC differentiation and matrix mineralization [192]. Additional factors—such as BMPs, transforming growth factor-β (TGF-β), and vascular endothelial growth factor (VEGF)—collectively enhance osteogenesis and tissue vascularization [193].

Combining PRP with autologous bone grafts or biomaterial scaffolds has shown promising results in bone regeneration, enhancing bioactivity, growth factor delivery, and cost-effectiveness [194,195]. Emerging strategies in PRP-based tissue engineering, which integrate scaffolds, MSCs, and platelet-derived growth factors, provide a biocompatible and biodegradable platform that enhances both bone and cartilage regeneration [192,196,197,198]. Owing to their multipotent differentiation capacity and broad tissue availability, MSCs represent an ideal cell source for osteogenic induction. Collectively, findings from clinical and in vitro studies indicate that PRP enhances osteogenic differentiation, increases mechanical strength, and accelerates fracture healing with minimal immunogenicity, underscoring its potential as a safe and effective adjunct in regenerative orthopedics [197,199,200].

#### 6.4.2. Ligament, Muscle, and Tendon Injury

Tendon injuries are common in sports and orthopedic practice and typically result from repetitive mechanical overloading. Their healing potential is limited by poor vascularity and low intrinsic regenerative capacity [201].

Tendon stem cells (TSCs) play a central role in tendon repair through their ability to proliferate and differentiate into tenocytes, thereby contributing to matrix synthesis and structural restoration. Studies have shown that activated PRP enhances TSC proliferation and collagen production [201]. Kim et al. demonstrated that the integration of bone marrow aspirate concentrate (BMAC), PRP, and tendon-derived stem cells (TDSCs) synergistically promoted cell proliferation and migration while preventing non-tenogenic differentiation in rotator cuff models [202]. However, Zhang et al. found that leukocyte-rich PRP reduced TSC proliferation but increased collagen production and tendon-like tissue formation compared to pure PRP [203]. Nevertheless, leukocyte-rich PRP also induced higher levels of inflammation, nontenocyte differentiation, and apoptosis [204]. Furthermore, differences in PRP activation methods—such as calcium chloride, thrombin, or mechanical activation—significantly influence TSC behavior and the overall quality of tendon repair, underscoring the importance of standardized PRP preparation protocols [201].

Clinical studies assessing PRP in tendon, ligament, and muscle injuries have yielded inconsistent outcomes. Some studies have reported objective improvements, such as MRI-confirmed healing of medial collateral ligament injuries [205] and a high return-to-sport rate (88%) following ulnar collateral ligament treatment [206]. However, other studies found limited or transient benefits For Achilles tendinopathy, PRP increased tendon thickness but did not improve functional scores [207]. In rotator cuff repair, PRP altered tissue properties but showed no long-term clinical benefit [208,209]. Overall, evidence supporting PRP’s clinical effectiveness remains inconclusive.

#### 6.4.3. Peripheral Nerve Injury

Peripheral nerve injuries (PNIs) present a significant clinical challenge due to limited regenerative capacity and frequent long-term disability, despite the intrinsic ability of axons to regenerate [210]. The current gold standard treatment—autologous nerve grafting—is effective but constrained by donor site morbidity, limited tissue availability, and mismatch in nerve length or diameter [211]. Acellular nerve allografts have achieved partial success in short-gap nerve repairs but their efficacy diminishes in large-gap defects due to insufficient cellular and trophic support [212]. To overcome these limitations, tissue-engineered nerve conduits have been developed to guide axonal growth and create a regenerative microenvironment; however, complications such as extrusion and fistula formation have been reported in the use of synthetic conduits for longer nerve gaps [213,214].

In this context, platelet-rich plasma (PRP) has emerged as a promising bioactive scaffold component in peripheral nerve regeneration. PRP provides a biomimetic and neurotrophic microenvironment that supports axonal outgrowth, angiogenesis, and Schwann cell proliferation and differentiation [215]. An ideal nerve scaffold should deliver both biochemical and structural cues—including collagen, laminin, and fibronectin—while maintaining porosity for oxygen diffusion, exhibiting low immunogenicity, and ensuring complete biodegradability [216]. The regenerative potential of PRP is largely attributed to its rich content of bioactive molecules, including transforming growth factor-β1 (TGF-β1), insulin-like growth factor-1 (IGF-1), platelet-derived growth factor-AB (PDGF-AB), vascular endothelial growth factor (VEGF), glial cell line-derived neurotrophic factor (GDNF), nerve growth factor (NGF), and exosome-associated microRNAs, which collectively enhance neurogenesis, angiogenesis, and cellular proliferation within the injured nerve milieu [203,217]. Preclinical and clinical data support PRP’s neuroregenerative potential. A placebo-controlled trial demonstrated significant symptom and sensory improvement after a single ultrasound-guided PRP injection in patients with carpal tunnel syndrome, indicating its potential role in promoting peripheral nerve repair [218]. Nonetheless, heterogeneity in PRP preparations—such as the use of platelet-rich fibrin—has been linked to inconsistent outcomes, including reduced functional recovery in animal models of nerve injury [219].

#### 6.4.4. Articular Cartilage Lesions and Osteoarthritis (OA)

Articular cartilage lesions are frequently encountered in daily orthopedic practice and remain therapeutically challenging because of the limited regenerative capacity of hyaline cartilage, which is composed predominantly of extracellular matrix and lacks vascular and neural supply [220,221]. Disruption of synovial homeostasis—including alterations in synovial fluid composition and inflammation—impairs cartilage repair and initiates a cascade of degenerative processes such as chondrocyte apoptosis, meniscal degeneration, subchondral sclerosis, and bone remodeling, ultimately leading to osteoarthritis (OA) [222,223,224]. Clinically, OA manifests as symptoms of joint pain, stiffness, and restricted mobility. Current treatments such as hyaluronic acid injections and microfracture aim to relieve symptoms and promote cartilage repair, but their efficacy remains limited.

**Figure 4:**
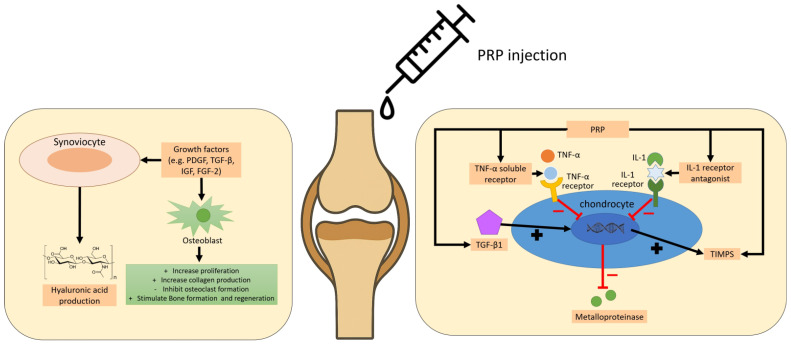
The molecular and cellular principle of PRP treatment in articular cartilage lesions and osteoarthritis.

The pathogenesis of OA involves a complex interplay of inflammatory mediators produced by the synovium and chondrocytes, with regional variability in cytokine expression patterns [225]. Intra-articular PRP injections have demonstrated anti-inflammatory effects by reducing synovial inflammation and downregulating proinflammatory cytokines—most notably interleukin-1β (IL-1β) and tumor necrosis factor-alpha (TNF-α)—thereby contributing to pain alleviation and cartilage preservation in both preclinical and clinical studies [226]. Figure 4 illustrates the molecular and cellular principle of PRP treatment in articular cartilage lesions and osteoarthritis. While some studies reported significant clinical improvement following PRP treatment, such as enhanced functional scores and sport activity levels [227], others found no superiority over hyaluronic acid (HA) or no correlation with patient demographics or degeneration severity [228].

Despite the therapeutic potential of PRP, its rapid clearance from cartilage defect sites due to poor tissue adhesion limits its residence time and clinical efficacy [229]. To address this, Liu et al. developed a photocross-linkable PRP-complexed hydrogel (HNPRP) that could enhance in situ gelation, growth factor retention, and cartilage integration, significantly improving repair outcomes in a rabbit model [230]. To overcome these limitations, PRP has been incorporated into various scaffolds, forming the foundation of PRP-based tissue-engineering strategies that integrate seed cells, growth factors, and biocompatible scaffolds to promote cartilage regeneration [231,232]. For instance, Yanasse et al. achieved successful cartilage repair using PRP scaffolds combined with human dental pulp stem cells in vivo [233].

Biomaterials designed for osteochondral defect repair must support cell migration and possess biodegradability, mechanical integrity, and biocompatibility. Chang et al. reported that combining a polylactic-coglycolic acid scaffold with PRP and passive motion therapy promoted hyaline cartilage and subchondral bone regeneration with limited inflammation [234]. Advances in 3D bioprinting further enable the fabrication of complex, multilayered scaffolds incorporating diverse cell types and materials in anatomically relevant configurations. However, challenges remain in maintaining cell viability and bioactivity while achieving mechanical stability and precise spatial distribution of cells and biomolecules, underscoring the need for further research [231].

## 7. Discussion

The clinical use of PRP has expanded considerably over the past two decades, yet significant heterogeneity remains regarding its definition, classification, and preparation. PRP is broadly characterized as an autologous blood derivative with a supra-physiological concentration of platelets, but its biological activity is influenced by leukocyte content, fibrin architecture, and activation methods. This variability has led to the development of multiple classification systems, each addressing different technical and biological parameters. The Dohan-Ehrenfest classification provided an initial framework by distinguishing PRP products according to leukocyte content and fibrin structure. Subsequent systems, such as PAW, PLRA, DEPA, and MARSPILL, have progressively integrated more refined parameters, including platelet dose, activation strategies, and cellular composition, reflecting the evolving complexity of PRP formulations. More recent coding systems aim to standardize PRP characterization for both clinical and research applications.

Preparation methods further contribute to heterogeneity, with double-spin and buffy coat protocols yielding distinct platelet and leukocyte profiles. While the double-spin method offers greater control over platelet concentration, the buffy coat technique inherently enriches leukocytes, making each method suitable for different therapeutic goals. The debate surrounding exogenous platelet activation highlights the need to balance immediate growth factor release with sustained bioactivity at the injury site. Heterogeneity in preparation methods—such as variations in platelet and leukocyte concentration—continues to limit reproducibility.

Horizontal and fixed-angle centrifugation are two types of rotor designs used in centrifuges, differing in how the tubes are held during operation. Horizontal rotors hold tubes in a “swing-out” position, aligning them perpendicular to the axis of rotation to create a flat, horizontal layer separation. Fixed-angle rotors hold tubes at a set angle, causing particles to sediment against the tube wall, which can be faster but may result in a less uniform pellet. To improve PRP production, it is better to use a double centrifugation method with specific speed and time parameters, a swing-out (horizontal) rotor, and a stable, vibration-free surface. The two-spin process involves a lower speed spin to separate plasma from red blood cells, followed by a higher speed spin to concentrate the platelets. 

To avoid centrifuge tube contamination, it is important to maintain aseptic technique by working in a clean environment, using personal protective equipment like sterile gloves, and sterilizing equipment and tubes. Prevent contamination during use by not overfilling tubes, ensuring proper sealing, avoiding contact with tube interiors, and using sterile pipettes. After centrifugation, decontaminate any spilled samples or contaminated equipment before proceeding. 

At the molecular level, PRP’s therapeutic potential stems from a rich repertoire of bioactive mediators, including platelet-derived growth factors, cytokines, and extracellular vesicles. The roles of leukocytes, plasma proteins, and residual red blood cells further influence regenerative outcomes, with leukocytes providing immune modulation and plasma proteins supporting stromal repair. However, excessive leukocyte or RBC content may exacerbate inflammation or tissue damage. Thus, optimizing PRP composition remains critical for maximizing efficacy across diverse clinical indications. Standardization of preparation and classification will be essential to ensure reproducibility, comparability, and wider clinical adoption.

PRP demonstrates a multifaceted role in tissue repair and regeneration, exerting effects that extend beyond its well-recognized capacity to release growth factors. Platelet adhesion molecules, including integrins and selectins, initiate and stabilize platelet–leukocyte and platelet–endothelial interactions, linking vascular injury with immune activation. These adhesion-dependent processes not only facilitate hemostasis but also drive immune cell recruitment, thereby bridging wound repair with immunomodulation. Importantly, PRP supports anti-inflammatory pathways, reducing key cytokines such as IL-1, IL-6, and TNF-α while promoting M2 macrophage polarization and regulatory T-cell activation. This dual capacity to stimulate repair while tempering inflammation highlights its unique therapeutic balance.

The immunomodulatory effects of PRP are further underscored by its influence on both innate and adaptive immunity. Platelet-derived CD40L and serotonin (5-HT) serve as critical mediators, enhancing T- and B-cell function while modulating dendritic cell activity. Such effects illustrate PRP’s potential to orchestrate an integrated immune response that supports tissue recovery. Angiogenesis is another vital outcome, largely mediated by VEGF and PDGF-BB, though dependent on optimal platelet concentration, with excessively high levels paradoxically impairing vascular formation. In parallel, PRP demonstrates analgesic potential, particularly through serotonin release, though clinical outcomes remain heterogeneous and mechanistic understanding incomplete.

Collectively, these findings suggest that PRP operates through a complex interplay of adhesion molecules, immunomodulation, angiogenesis, and nociceptive regulation. However, variability in PRP preparation—such as platelet concentration, leukocyte content, and activation method—remains a critical limitation in standardizing outcomes. Further mechanistic studies and controlled clinical trials are warranted to refine protocols, optimize dosing, and clarify patient-specific responses. By integrating its regenerative, anti-inflammatory, immunomodulatory, angiogenic, and analgesic properties, PRP holds significant promise as a versatile therapeutic strategy across diverse clinical settings.

These interwoven mechanisms explain why PRP has diverse applications across a broad spectrum of medical fields. In orthopedics, its angiogenic and immunoregulatory effects contribute to tendon, cartilage, and bone healing. In dermatology and aesthetics, its ability to stimulate angiogenesis and dermal remodeling supports skin rejuvenation and hair restoration therapies. In dentistry and oral surgery, PRP accelerates wound healing and osseointegration, while in wound care it reduces inflammation and enhances granulation tissue formation. Overall, PRP represents a biologically active, versatile therapy whose mechanistic complexity directly underpins its wide-ranging clinical applications.

PRP (Platelet-Rich Plasma) and PRF (Platelet-Rich Fibrin) are both blood-derived treatments that use the body’s own fluid components and healing factors to promote tissue repair and rejuvenation, but they differ in their processing and results. PRF is a “next generation” therapy that uses a slower centrifuge speed to create a fibrin matrix scaffold with network structure, resulting in a more gradual release of growth factors for longlasting effects. PRP is processed faster and releases growth factors more quickly, often requiring anticoagulants, while PRF is prepared without additives and has a higher concentration of white blood cells and platelets. Therefore, PRF can serve as a second-generation platelet concentrate to exert longer and stronger effects on the human body.

Based on the principle mentioned above, liquid injectable Platelet-Rich Fibrin (i-PRF) is a second-generation regenerative medicine product derived from a patient’s own blood that stays liquid for a short period before turning into a gel. It is used for its ability to promote tissue regeneration, reduce inflammation, and accelerate healing in areas like orthopedics, dermatology, and wound healing. The process involves drawing blood, using a special low-speed centrifugation to create a liquid concentrate of platelets, fibrin, and white blood cells, and then injecting it into the treatment area.

## 8. Conclusions

Platelet-rich plasma (PRP) is an autologous blood product enriched with platelets and bioactive mediators that promote tissue repair. Variability in its preparation and composition has led to the development of multiple classification systems, including Dohan-Ehrenfest, PAW, PLRA, DEPA, MARSPILL, and the six-digit coding model. Preparation methods, such as double-spin and buffy coat techniques, influence platelet and leukocyte concentrations, impacting therapeutic outcomes. PRP contains platelets, leukocytes, plasma proteins, and exosomes that modulate regeneration. Standardized classification and optimized preparation remain essential for consistent clinical efficacy.

PRP promotes tissue repair and healing through coordinated mechanisms involving platelet adhesion molecules, immune modulation, angiogenesis, and pain regulation, offering a multifactorial basis for its therapeutic effects. It reduces inflammation, enhances M2 macrophage activity, and supports both innate and adaptive immune responses, while growth factors like VEGF and PDGF-BB stimulate vascular regeneration. Platelet-derived serotonin further contributes to analgesic effects. These mechanisms translate into diverse applications, including musculoskeletal repair, dermatology, gynecology, dentistry, and wound management. PRP enhances tissue regeneration while modulating inflammation and pain, though clinical outcomes remain variable due to preparation differences. Despite its therapeutic potential, variability in PRP preparation significantly impacts outcomes, highlighting the need for standardized protocols. Overall, PRP offers a multifactorial and promising approach for regenerative medicine.

## Figures and Tables

**Table 1:** The classification of PRP.

Classification System	Criteria	Subtypes/Levels	Definition and Features
Ehrenfest (2009) [1]	Leukocyte content and fibrin architecture	P-PRP (Pure Platelet-Rich Plasma) L-PRP (Leukocyte- and Platelet-Rich Plasma) P-PRF (Pure Platelet-Rich Fibrin) L-PRF (Leukocyte- and Platelet-Rich Fibrin)	- P-PRP: Low leukocytes, liquid form. - L-PRP: High leukocytes, liquid form. - P-PRF/L-PRF: Solid/gel form, used as a membrane. - Does not quantify platelet count.
PAW Classification (2012) [2]	Platelet count, Activation status, WBC content	P1–P4: Platelet concentration A+/A−: With/without exogenous activation W0–W3: WBC level and subtype (e.g., W3 includes neutrophils)	Provides quantitative platelet levels. Considers activation methods. Specifies WBC subtypes (neutrophils).
PLRA Classification (2015) [3]	Platelet concentration, Leukocyte content, RBC presence, Activation method	e.g., P3-L2-R1-A+	- P: Platelet count (1–4× baseline) - L: Leukocytes (0 = absent, 1 = low, 2 = high) - R: RBCs (0 = none, 1 = present) - A: Activation (±)
DEPA Classification (2016) [4]	Dose, Efficiency, Purity, Activation	- Dose: Total number of platelets injected - Efficiency: % of platelets recovered from whole blood - Purity: Ratio of platelets to RBCs and leukocytes - Activation: A+ or A−	- Focuses on quality of PRP preparation. - Encourages standardized reporting of platelet dose. - Ignores WBC subtypes.
MARSPILL (2020) [5]	Method, Activation, RBCs, Spin, Platelet conc., Image guidance, Leukocytes, Light activation	Each letter is a reporting item	- M: Preparation method (e.g., manual vs. kit) - A: Activation method - R: RBC presence - S: Centrifugation details - P: Platelet concentration - I: Image guidance - L: Leukocyte presence - L: Light therapy use
Six-Digit Coding System (2020) [6]	platelet composition, purity, and activation	N1–N6	- N1: Basal platelet concentration in blood - N2: Platelet concentration in PRP - N3: Red Blood Cells in PRP - N4: White Blood Cells in PRP - N5: External Activation - N6: Calcium Addition

**Table 2:** A summary of bioactive molecules in platelets.

Component	Size/Structure	Key Contents	Primary Functions
α-Granules	300–500 nm membrane-bound vesicles; 50–60 per platelet	Growth factors (PDGF, TGF-β1, VEGF, bFGF, EGF), adhesive proteins, coagulation, fibrinolytic factors and others listed in Table 2.	Regulate tissue repair, hemostasis, angiogenesis, immune modulation
Platelet-Derived Exosomes	30–150 nm vesicles derived from multivesicular bodies	Proteins, mRNA, miRNA, lipids; growth factors encapsulated (PDGF, VEGF, TGF-β1)	Mediators of intercellular communication and delivery of regenerative signals
Dense Granules (δ-granules)	250–300 nm organelles	ADP, ATP, serotonin (5-HT), calcium, histamine, epinephrine, polyphosphates	Promote platelet activation, vasoconstriction, and immune signaling
Lysosomes (λ-granules)	50 to 500 nm organelles	Acid hydrolases (collagenase, elastase, cathepsins), antimicrobial proteases	Contribute to ECM remodeling, fibrinolysis, antimicrobial defense

**Table 3:** Bioactive proteins and growth factors released from α-granule.

Structure	Key Content	Main Functions
α-granules	Growth Factors: PDGF (AA-BB-AB-CC), VEGF, TGF (α-β), FGF (a-b), EGF, CTGF	Cell proliferation, chemotaxis, angiogenesis, collagen synthesis, and tissue remodeling
	Adhesive Proteins: Fibronectin, vitronectin, fibrinogen, vWF, P-selectin, integrins αIIbβ, Phosphatidylserine	Platelet aggregation, platelet–endothelial cell interaction, and thrombus formation.
	Coagulation Factors: Factors IV, XI, XIII, plasminogen, plasmin, antithrombin, tissue factor	Initiate and regulate clot formation, stabilize fibrin networks, and support clot breakdown to maintain hemostatic balance
	Angiogenic Regulators: IL8, thrombospondin, Angiostatin, PF-4, TIMP-1,4, MMP-1,2,9, Angiopoietin, Endostatin, SDF-1, PMP	Angiogenesis cascades, coordinate blood vessel formation, remodeling, and stabilization to restore tissue perfusion and support regenerative processes.
	Cytokines: IL1, IL4, IL6, TFNα, SDF-1	Chemotaxis, inflammatory response modulation, and antimicrobial activity.
	Chemokines: RANTES, CXCL4, CXCL7, CCL2, CCL3, CCL5, β-TG	Inflammation, antimicrobial, and bactericidal activity.
	Complement Proteins: C3, C4	Phagocytosis, chemotaxis, and platelet activation.
	Exosomes: mRNA, miRNA, CXCL4, CXCL7	Cell adhesion, paracrine communication, regulation of cell fate, and promote tissue regeneration by delivering growth factors, RNAs, and signaling molecules to target cells.

## Data Availability

No new data were created or analyzed in this study. Data sharing is not applicable to this article.
